# Turning over New Leaves

**Published:** 1890-08-16

**Authors:** 


### Turning Over New Leaves.

**"Hospitals" in Chambers' Encyclopædia.**

We have received Volume V. of "Chambers' Encyclopædia," new edition. Having had the older edition in our library for many years, we are disappointed to find that the section on hospitals has evidently been placed in the hands of a novice, or, at any rate, of someone who has little practical acquaintance with the subject.

The section omits all mention of the historical aspect of the question—a serious omission, and one, withal, so important as to make the article relatively valueless. From what is said about construction, we should gather that the writer has had more familiarity with workhouses than with asylums. It is remarkable that he should state that from four to eight patients are the maximum number for the circular form of ward, whereas it is well known that a circular ward is comparatively ill-adapted for so small a number of beds, as experience has now proved. The reproduction of this old view goes to prove that the article is out of date and behind the times. This is more strikingly illustrated by the following statement: "The first principle of the ward unit is that the ward and ward offices should be self-contained within one door, commanded by the head nurse's room, so that at any moment she may know where every patient is."

Now if there is one thing more condemned than another by the best administrators it is the peep-hole system here recommended. This system has been given up for years on the principle that a well-regulated hospital must work like a machine where each part fits so exactly into its fellow that the whole mechanism runs smoothly and without friction. Where these principles are understood and enforced espionage is not only unnecessary, but held in deserved contempt. It might be thought, however, from the article in "Chambers" that no British hospital could be conducted without some such vicious system. Again, here is an extraordinary definition: "Nursing is putting us in the best possible conditions for nature to restore or to preserve health—to prevent or to cure disease or injury." Perhaps some reader will explain the meaning of this sentence, which is not too clear on the face of it.

The same incompleteness and skimpiness is observable throughout, and it would scarcely be believed that no allusion is made to cottage hospitals, pay hospitals, or fever hospitals, or to Hospital Saturday, to mention only a few of the omissions; while the bibliography is so meagre as to give special prominence to the works of one man who is not an authority on the subject, he having had no practical experience of hospitals whatever. We hope Messrs. Chambers will take steps to have this article re-written and brought up to date, as it at present constitutes a serious blot on a most useful and helpful publication.

